# Book Reviews

**Published:** 1902-07

**Authors:** 


### BOOK REVIEWS.

NEUROSES OF THE GENITO-URINARY SYSTEM IN THE MALE, WITH STERILITY AND IMPOTENCE. By Dr. R. Ultzmann, Professor of Genito-urinary Diseases in the University of Vienna. Second Edition, Revised, with Notes and a Supplementary Article on Nervous Impotence by the Translator, Gardner W. Allen, M.D., Surgeon in the Genito-urinary Department of the Boston Dispensary; Instructor of Genito-urinary Surgery in Tuft's Medical College. Illustrated. Philadelphia: F. A. Davis Co.

Ultzmann's name has been long associated with genito-urinary diseases and this translation of his recent work will arouse immediate interest. The book is a collection of studies on neuroses of the genito-urinary apparatus—a subject of great moment to the practitioner, for it deals with a very perplexing phase of medicine and additional light is exceedingly welcome. The translation is admirably done.

---

PRACTICAL MEDICINE SERIES. MAY, 1902. GENERAL MEDICINE.

This volume contains the gist of opinion formulated to date of publication on the following subjects: Typhoid fever, malaria, dysentery, mountain fever and other fevers, peritonitis, diseases of the abdominal organs, diseases of the stomach and intestines. The volume is one of a series issued monthly, and is edited by Dr. Frank Billings. The publishers are The Year Book Publishers, 40 Dearborn street, Chicago. The price of this volume is $1.50, of the series $7.50.

---

PROGRESSIVE MEDICINE. JUNE, 1902.

This volume is concerned with the subjects of abdominal surgery, gynecology, diseases of the blood, diseases of the glandular and lymphatic systems, metabolic diseases and gynecology. This series is intended to cover the progress of medicine in its various departments, and is of great use in assisting the practitioner in keeping abreast of the times.

